# Mimicking Polymer Processing Conditions on the Meso-Scale: Relaxation and Crystallization in Polyethylene Systems after Uni- and Biaxial Stretching

**DOI:** 10.3390/molecules29143391

**Published:** 2024-07-18

**Authors:** Dirk Grommes, Olaf Bruch, Dirk Reith

**Affiliations:** 1 Institute of Technology, Resource and Energy-Efficient Engineering (TREE), Bonn-Rhein-Sieg University of Applied Sciences, Grantham-Allee 20, 53757 Sankt Augustin, Germany; olaf.bruch@h-brs.de (O.B.); dirk.reith@h-brs.de (D.R.); 2 Dr. Reinold Hagen Stiftung, Kautexstrasse 53, 53229 Bonn, Germany; 3 Fraunhofer Institute for Algorithms and Scientific Computing (SCAI), Schloss Birlinghoven, 53754 Sankt Augustin, Germany

**Keywords:** mesoscale coarse-graining, polyethylene, biaxial stretching, relaxation, crystallization, local chain orientation

## Abstract

Highly varying process conditions drive polymers into nonequilibrium molecular conformations. This has direct implications for the resulting structural and mechanical properties. This study rigorously investigated processing-property relations from a microscopic perspective. The corresponding models use a mesoscale molecular dynamics (MD) approach. Different loading conditions, including uniaxial and biaxial stretching, along with various cooling conditions, were employed to mimic process conditions on the micro-scale. The resulting intricate interplay between equi-biaxial stretching, orientation, and crystallization behavior in long polyethylene chains was reviewed. The study reveals notable effects depending on different cooling and biaxial stretching procedures. The findings emphasize the significance of considering distributions and directions of chain ordering. Local inspections of trajectories unveil that crystal growth predominantly occurs in regions devoid of entanglements.

## 1. Introduction

During the design process of plastic components, engineers face complex situations: not only the part itself but also the production process must be considered for the objective of an optimal product. As semi-crystalline polymers significantly change their properties during processing, especially under the occurrence of flow fields or stretching processes [1,2,3,4], these changes need to be quantified. With respect to typical polymer processing techniques such as deep drawing, film blowing or extrusion blow molding stretching procedures as well as conditions during cooling have significant influence on the material and thus part properties [5,6,7,8,9]. Experimental results clearly show that the mechanical properties such as the elastic modulus depend on the level of stretching during processing [10,11]. Relaxation and corresponding shrinkage effects after cooling show similar dependencies [7,10]. Local orientations of chains and associated effects on crystal growth have been demonstrated [6,12,13,14]. Although these effects are well documented, experiments do not give full insights into the dynamics that happen under processing conditions [15]. The underlying micro-structural phenomena are experimentally not accessible.

Molecular dynamics (MD) simulation models are, therefore, a good starting point for a deeper understanding of mechanisms that drive the polymer’s behavior under real-life processing conditions. MD simulations of the crystallization behavior of priorly stretched polyethylene systems are found in [16,17,18,19,20,21,22,23]. The focus of these studies is on uniaxially stretched or sheared systems. Investigations of biaxially stretched systems are found in [24]. In contrast to our work, [24] used a constant total level of stretching for all investigations ($\lambda_{\mathrm{biaxial}}=9$ according to the definition in [24]). They reported a significant influence of the different stretch ratios on orientation and crystallization behaviors. Also, Zhang et al. [25] investigated uni- and biaxially stretched polyethylene systems. Their focus was on the influence of different stretching approaches on stress–strain properties and failure behavior. Hall et al. [26] used a coarse-grained polyethylene model (mapping of three CH2 units into one superbead), which resembles the model used in our study. They investigated various static and dynamic properties of polyethylene, including its crystallization behavior from quiescent melt. Studies specifically examining the influence of entanglements on the crystallization behavior of polymers have been published by Zou et al. [27] and Zhu et al. [28]. A comparison of the crystallization behavior of polyethylene by the use of different united atom force fields is found in Hagita et al. [29]. Concerning underlying theories, it is well-established that flow-enhanced nucleation (FEN) occurs during the stretching of polymer melts, as explained by Flory’s conformational entropy reduction model (CERM) [30].

In preceding contributions [22,31], we investigated the behavior of coarse-grained polyethylene systems under different uni- and biaxial stretching processes. By using MD methods, relationships between the micro-mechanical state after stretching and the following initial crystallization behavior have been determined. Since the studied time span in a previous work [31] was only 10 ns, which is very short, the question of how these systems develop over a longer period remains unanswered. Therefore, in this work, the evolution of these systems is examined over an extended time span of up to 200 ns. As in the previous publication, the approach of using boundary conditions that mimic those found in real-life processes is maintained. In this context, we basically try to mimic conditions found in the extrusion blow molding process. In contrast to other studies, our research describes the entire loading history of the polyethylene melt from the stretching (blowing process) through cooling (under mold constraint or free conditions) to the subsequent relaxation process after demolding of the part. All of this is examined purely on the micro-scale by using coarse-grained MD simulations. The main focus of the study is on the system behavior after cooling from the melt state (500 K) to room temperature (293.15 K) is finished. The impact of different uni- and biaxially stretch ratios on the relaxation and crystallization behavior of the polymer systems is examined. In addition, the influence of different cooling procedures on the microscopic structure of the polyethylene systems is evaluated. These conditions mimic the effect of mold constraints during cooling, corresponding to typical conditions in the extrusion blow molding process. For the purpose of comparison, systems are also cooled under free conditions without any mechanical constraints. Additionally, cooling times are varied. The evolution of the investigated systems is evaluated by the analysis of microscopic observables such as the orientations of chain segments, nematic ordering, number and location of entanglements, and the level of crystallinity.

## 2. Results

In the following subsections, the effects that occur in priorly stretched and cooled polyethylene systems are described. The presentation of results starts by reporting the general system behavior depending on different stretching levels and the chain length. Subsequently, the influence of varying boundary conditions and cooling time is discussed.

### 2.1. General Crystallization and Relaxation Behavior

**Figure 1:**
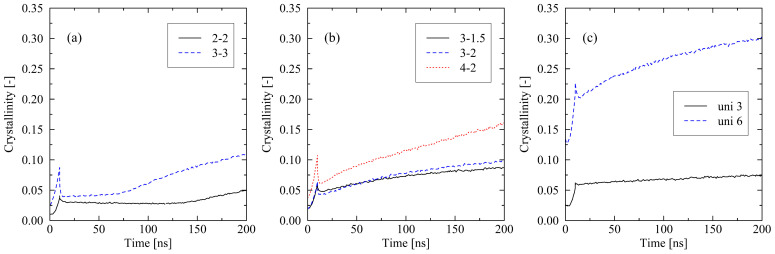
Crystallinity plotted over time for different stretching procedures ((**a**) equi-biaxially stretched systems, (**b**) biaxially stretched systems, (**c**) uniaxially stretched systems). The systems (size: 250×2000 (M×N)) were subject to fixed boundary conditions during cooling. The figures include the system behavior during cooling from 500 K to 293.15 K (0…10 ns).

First, how the investigated systems crystallize after the cooling finishes is evaluated. We describe the behavior of systems that are subject to fixed boundary conditions during cooling. From Figure 1, it is seen that during the cooling stage (0…10 ns), crystal growth is initialized. The formation of crystals during that stage is described in our previous publication [31]. At 10 ns, a strong decrease in the level of crystallinity is obvious. This is caused by the release of the fixed boundary conditions after the cooling phase. From that point on, systems can change their shape and size. Subsequently, we monitor distinctively different system behaviors, which strongly depend on the level of (biaxial) stretching. For systems that were previously stretched by the use of a dominating main direction (3-1.5, 3-2, 4-2), we report immediate (strong) increasing levels of crystallization after release (10 ns). In the case of the uniaxially stretched systems, a slow but constantly increasing level of crystallinity for the uni 3 system and strong crystallization for the uni 6 system is obvious. The equi-biaxially stretched systems develop quite differently. For a significant period of time, these systems tend not to or only very slowly crystallize. After a certain reorganization phase is passed, the systems start to rapidly crystallize (2-2: t>140ns; 3-3: t>70ns).

**Figure 2:**
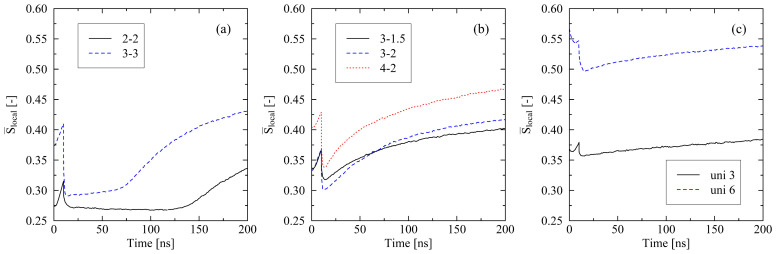
Nematic order parameter ${\bar{S}}_{\mathrm{local}}$ plotted over time for different stretching procedures ((**a**) equi-biaxially stretched systems, (**b**) biaxially stretched systems, (**c**) uniaxially stretched systems). Systems (size: 250×2000 (M×N)) are subject to fixed boundary conditions during cooling. Figures include the system behavior during cooling from 500 K to 293.15 K (0…10 ns).

The differently monitored crystallization behavior can be explained by the interplay of local ordering of chain segments and their entanglements. Concerning the local chain ordering, evaluated by the local order parameter ${\bar{S}}_{\mathrm{local}}$ (Figure 2), we report that the initial loss of local ordering is generally larger for higher levels of stretching. The loss of order is most notable in the case of the widely stretched 3-3 system.

**Figure 3:**
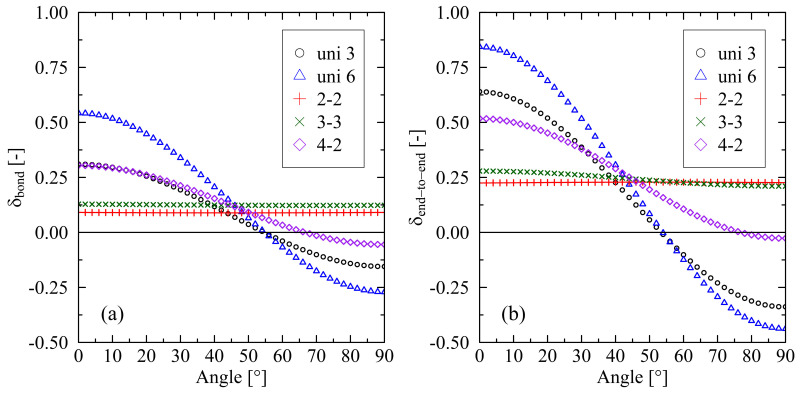
Orientation of local bond vectors $\delta_{\mathrm{bond}}$ (**a**) and chain end-to-end vectors $\delta_{\mathrm{end}-\mathrm{to}-\mathrm{end}}$ (**b**) plotted over different testing angles within the stretching plane. The figure shows data obtained at the end of the stretching process. An angle of 0° corresponds to the main stretching direction, whereas an angle of 90° belongs to the minor stretching direction. According to the definition in Equation (1), a value of 1.0 represents full orientation in the corresponding direction, whereas a value of 0 implies isotropic distribution with respect to the testing angle. A value of −0.5 indicates full orientation in the perpendicular direction. The system size is 250×2000 (M×N).

A deeper inspection of the 3-3 (and analogously of the 2-2) system reveals that the orientation of local bond vectors $\delta_{\mathrm{bond}}$ as well as chain end-to-end vectors $\delta_{\mathrm{end}-\mathrm{to}-\mathrm{end}}$ is almost evenly distributed within the stretching plane (Figure 3). In contrast, systems with a predominant stretching direction clearly exhibit most orientations aligned with this dominating direction. As a consequence, initially, there is no clearly preferred direction for the formation of crystalline zones in the equi-biaxially stretched systems. From a micro-mechanical perspective, this specific orientation behavior is the reason for the monitored delay until chain segments enter a (local) well-parallel ordered state. Only after this reorganization phase does fast formation of crystallites set in (Figure 1a). For all investigated systems, it applies that they tend to reorder after they initially lose their well-ordered state. The ordering of chain segments is a prerequisite for the forming of crystallites, e.g., for the 2-2 system, local reordering starts at about 120 ns, which results in an increasing level of crystallinity after approx. 140 ns.

Connecting Figure 2 and Figure 3 reveals that the initial loss of nematic ordering after release at 10 ns correlates with the orientation of bond and chain end-to-end vectors. For comparable levels of stretching, the following applies: the stronger a preferred direction of orientations (Figure 3), the lesser the loss of nematic order at the end of the cooling process when mechanical constraints are released (Figure 2 (10 ns)). This suggests that relaxation processes with respect to local order are more pronounced in biaxially stretched systems than in uniaxially stretched systems.

**Figure 4:**
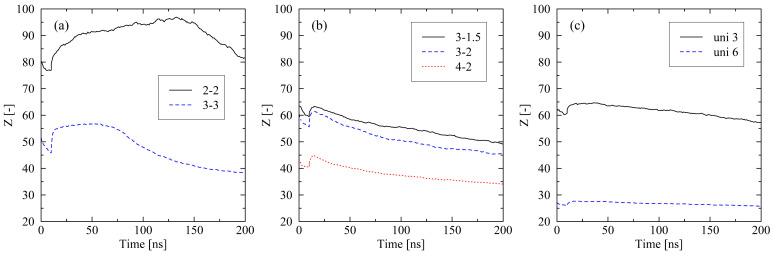
Number of entanglements *Z* per chain plotted over time for different stretching procedures ((**a**) equi-biaxially stretched systems, (**b**) biaxially stretched systems, (**c**) uniaxially stretched systems). The systems (size: 250×2000 (M×N)) were subject to fixed boundary conditions during cooling. The figures include the system behavior during cooling from 500 K to 293.15 K (0…10 ns).

**Figure 5:**
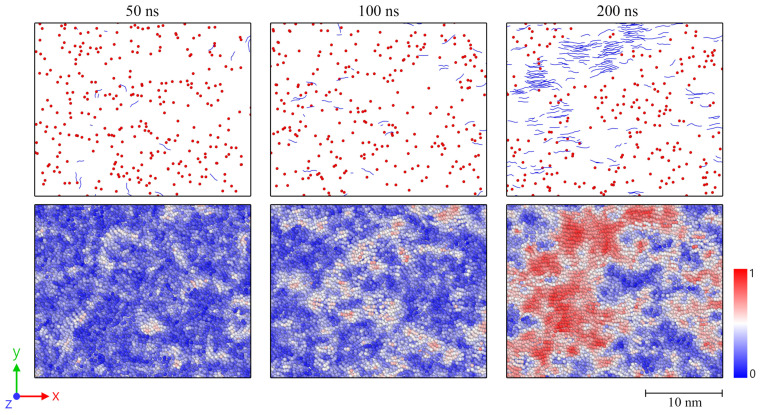
Snapshots of an equi-biaxially stretched (3-3) system with size 250×2000 (M×N) after 50, 100, and 200 ns simulation times, respectively. The snapshots are representative cut-outs from the larger system with a slice thickness of 2 nm (z-direction). The first row displays entanglement points of chains (red dots) and crystalline chain segments (blue lines) according to the definitions in Section 4 (with stem size $N_{\mathrm{stem}}\geq 5$). For reasons of clarity, amorphous regions are not displayed. The second row shows the nematic order parameter calculated for each bead within the cut-out segment. The color scale ranges from 0 to 1, where 1 (red color) represents ideal local alignment of chain segments. Figures were plotted by use of the OVITO visualization package (version 3.7.11) [32].

Interestingly, the level of nematic ordering for the 4-2 system after release falls below the corresponding value of the uni 3 system (${\bar{S}}_{\mathrm{local},\;4-2}=0.337$, ${\bar{S}}_{\mathrm{local},\;\mathrm{uni}\;3}=0.357$). Afterward, local ordering, as well as the level of crystallinity, increases considerably faster within the 4-2 system. This observation makes it clear that besides the ordering of chain segments, other micro-structural effects have a crucial impact on the crystallization behavior. In the specific case, entanglements of chains play a dominating role. Figure 4 shows that the number of entanglements per chain of the 4-2 system is far below the corresponding value of the uni 3 system. This is because the total level of stretching in the 4-2 system is significantly higher than in the uni 3 system [31], which results in more disentanglements of chains. It follows that the 4-2 system, despite a lower level of local chain ordering, rapidly crystallizes over time. From Figure 4, it is likewise clear that crystallization is connected to a decrease in the number of entanglements. Systematically, all systems disentangle to an increasing extent when crystallization processes occur. However, this continuous disentanglement process during crystallization is low and not the driving force of crystallization, but rather a side effect. The crucial factor for the further crystallization behavior is the increase in the local order of chain segments (Figure 2). Figure 5 gives an insight into the local formation of well-ordered regions and the resulting development of crystalline structures. While at 50 and 100 ns, respectively, only few and small crystals are visible; at 200 ns, larger crystals are monitored. Additionally, a strong increase in local orientations, as a precursor to the formation of crystals, is observed (Figure 5, color plot). From the figure, it becomes finally clear that crystal growth takes place in areas where almost any entanglement points exist.

**Figure 6:**
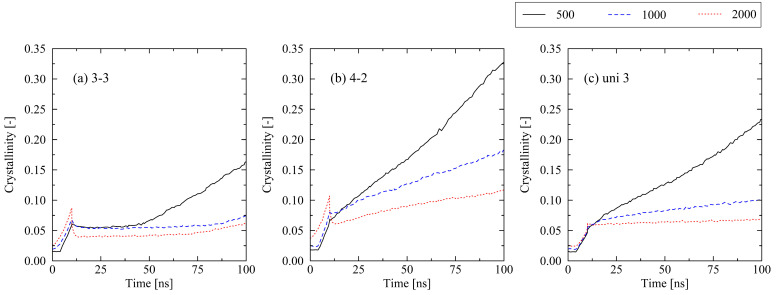
Influence of chain length (N=500,1000,2000) on the crystallization behavior of differently stretched systems ((**a**) equi-biaxial, (**b**) biaxial, (**c**) uniaxial). The systems were subject to fixed boundary conditions during cooling. The figures include the system behavior during cooling from 500 K to 293.15 K (0…10 ns).

With respect to the effect of the chain length, we only present results that occur within a time frame of 100 ns. This specific span of time is sufficient for the identification of the general influence of the chain length on the results. Additionally, we limit the discussion to certain levels of stretching, which represent typically observed behavior. Monitored effects fall in line with our previous investigation of uniaxially stretched and relaxed systems in [22]: systems consisting of short chains crystallize faster than long-chain systems (Figure 6). From the results of the equi-biaxial systems (3-3), it is also obvious that crystallization starts earlier for short chains. As already extensively discussed in [31], these observations can be explained by the lower levels of entanglements per chain and strong orientations in systems with short chains. Hence, further discussion of the influence of the chain length is omitted in this work.

### 2.2. Influence of Varying Conditions at Cooling Stage

#### 2.2.1. Mechanical Boundary Conditions

**Figure 7:**
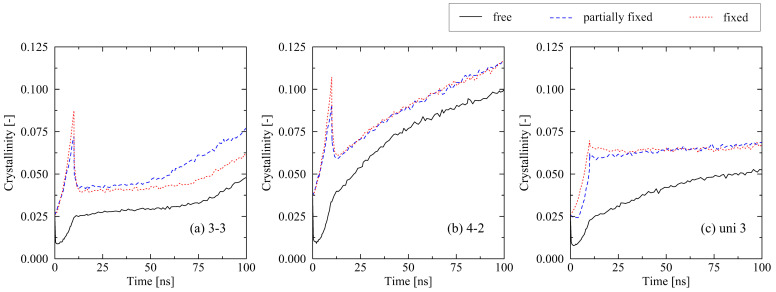
Influence of varying boundary conditions (free, partially fixed, fixed conditions) at the cooling stage (0…10 ns) on the crystallization behavior of differently stretched systems ((**a**) equi-biaxial, (**b**) biaxial, (**c**) uniaxial). The system size is 250×2000 (M×N).

We first compare the impact of free and fully fixed boundary conditions during cooling on the results. Findings from our uniaxial investigations [22] are also valid for the biaxially stretched systems: fixed conditions lead to a generally higher level of crystallization as chains are held in their stretched and oriented state during cooling (Figure 7). This strictly enforces the forming of crystalline structures. We report this behavior throughout all investigated levels of stretching.

In addition, we compare fully fixed and partially fixed conditions. In the latter case, the system is able to shrink in the perpendicular direction during cooling. In our previous studies, we assumed that it is valid to use the fully fixed condition for mimicking conditions during cooling under mold constraint [22,31]. Ideally, the real-life conditions correspond more to what is here described as partially fixed conditions. Therefore, it is highly important to verify whether there are substantial differences between the use of partially or fully fixed boundary conditions. Within the investigated cooling time, we report no essential differences between the discussed boundary conditions. Therefore, the investigations of fully fixed systems are regarded as applicable with respect to a comparison with real-life processing conditions. Nevertheless, in the case of the 3-3 system, a slightly earlier start of the fast crystallization regime at around 55 ns for the partially fixed system is obvious. As it is assumed that the exact point at which the equi-biaxially stretched systems start to pass over into the fast crystallization regime is subject to a certain statistical range, this effect needs further investigations in future publications.

#### 2.2.2. Cooling Time

**Figure 8:**
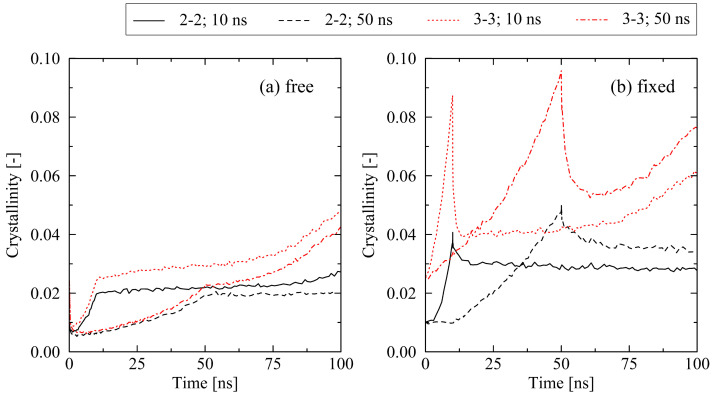
Influence of the cooling time (10 ns, 50 ns) on the crystallization behavior of equi-biaxially stretched systems (2-2, 3-3). A comparison of (**a**) free and (**b**) fixed conditions reveals a clear interaction effect of boundary conditions and cooling time.

**Figure 9:**
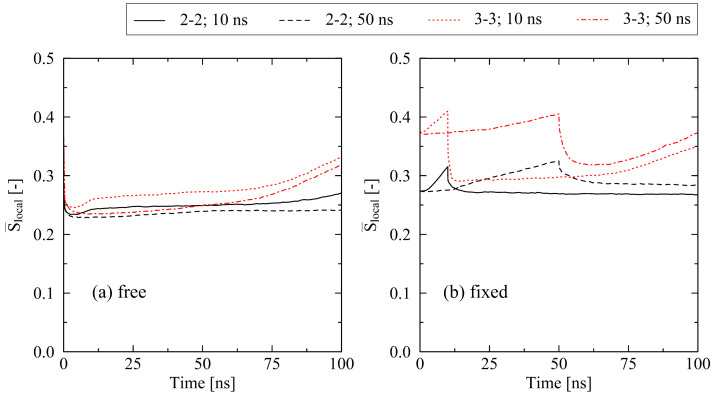
Evolution of the nematic order parameter ${\bar{S}}_{\mathrm{local}}$ depending on the boundary conditions ((**a**) free, (**b**) fixed), and cooling time (10 ns, 50 ns) for two different equi-biaxially stretched systems (2-2, 3-3).

Regarding the influence of the cooling time on the results, it is necessary to also take the mechanical boundary conditions during cooling into account. Results for the equi-biaxially stretched systems (2-2 and 3-3) show occurring effects very clearly: according to Figure 8, two scenarios exist, where the impact of the cooling time has opposite effects on the crystallization behavior. Under free conditions (Figure 8a), a short cooling time (10 ns) results in a higher level of crystallinity, whereas under fixed conditions, a long cooling time (50 ns) is favorable for higher levels of crystallinity (Figure 8b). This effect is explained by the relaxation behavior of chains. For free conditions, a slow cooling procedure gives chains the ability to quickly relax to amorphous structures as (1) instantaneous relaxation due to the early release of systems is enabled, and (2) systems are subject to the temperature range above the crystallization onset temperature ($T_{c,\mathrm{on}}\approx 375\;K$ [22]) for a longer period of time. In the case of fixed conditions, which hold chains to a significant degree in their well-oriented state from the stretching stage, a slow cooling results in additional time for the formation of highly parallel configurations and, hence, initial crystalline structures. Figure 9 supports these findings by showing the internal ordering of the systems by the nematic order parameter ${\bar{S}}_{\mathrm{local}}$. Additionally, in the case of fixed conditions, the level of internal stress at the end of the long cooling procedure is significantly lower compared to the short cooling procedure. This is a crucial factor, especially for highly stretched systems such as the 3-3 system. For this specific system, we observe a decrease in the internal stress (in stretching direction) at the end of the cooling from 41.9 N/mm² (10 ns cooling time) to 10.4 N/mm² (50 ns cooling time). For the lower stress case, the release of the system at the end of the cooling results in less micro-mechanically disturbing effects; hence, the monitored trend tends towards a stronger conservation of internal ordering as well as crystalline structures (Figure 8b and Figure 9b).

**Figure 10:**
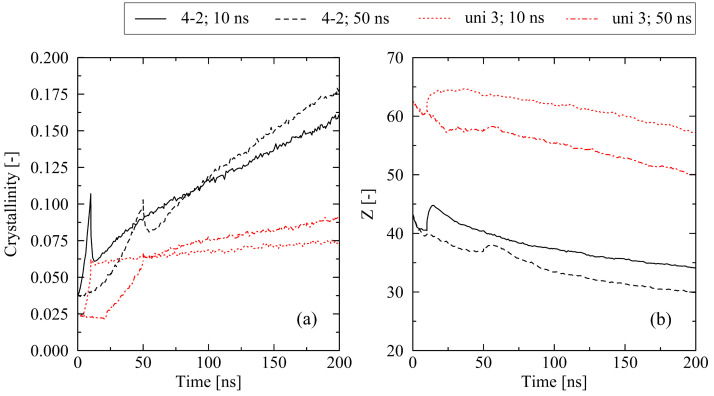
Crystallization behavior (**a**) and number of entanglements *Z* per chain (**b**) for bi- and uniaxially stretched systems (4-2, uni 3) depending on the cooling time (10 ns, 50 ns). The systems (250×2000 (M×N)) were subject to fixed boundary conditions during cooling.

For all other investigated levels of stretching (e.g., 4-2 and uni 3 (Figure 10)), the results display the same general trend as seen for equi-biaxially stretched systems. For fixed boundary conditions, a long cooling time is favorable for high levels of crystallinity, whereas in the case of free conditions, relations are reversed. However, for the fixed conditions, some additional effects must be noted. At the end of the cooling, the level of crystallinity for the 50 ns simulations falls below the corresponding value of the 10 ns cooling time simulations (Figure 10a). This effect occurs due to the missing reorganization phase in the non-equi-biaxially stretched systems (cf. Figure 1). Nevertheless, after the cooling stage is finished, all systems that have been subject to the long cooling time crystallize faster than the corresponding systems at short cooling times. This can be ascribed to a significantly less entangled state after cooling in the 50 ns systems (Figure 10b). As already demonstrated in Section 2.1 as well as in our previous work [22,31], a lower level of entanglements supports fast crystallization processes.

## 3. Discussion

We report remarkable effects with respect to equi-biaxially stretched systems. The monitored re-organization phase after the release of the systems (Figure 1 and Figure 2) emphasizes that it is not only the amount of mechanically induced orientations of chain segments that influences crystallization behavior. Also, local distributions (directions) of chain ordering must be taken into account. Our previous work [31] as well as [24] already provide hints towards the importance of a dominating stretching direction with respect to the fast growth of crystallites. By performing local inspections of trajectories, we precisely demonstrate that crystal growth predominantly takes place in regions of absence of entanglements (Figure 5). This behavior is consistent with the results observed by Zhu et al. [28] through MD simulations of cyclically stretched polymer systems. Clearly, orientations of chain segments within regions of absence of entanglements are the precursor for crystallization processes. From a global perspective, we were already able to reveal the general relation of entanglement length and resulting crystallinity during cooling processes [31]. Newly monitored chain length effects (Figure 6) are within the expected range [22,31].

Surprisingly, the effect of the newly introduced partially fixed boundary conditions is almost negligible (Figure 7). Within the investigated time frame (in particular with respect to cooling time), there is no crucial effect of shrinkage in thickness direction during cooling on the crystallization behavior. This gives rise to the conclusion that using fully fixed conditions, as we previously presumed in [22,31], is a viable assumption for the comparison with real-life conditions.

In contrast, we report a two-way interaction of cooling time and free or fixed boundary conditions, respectively. While at free conditions, a short cooling time results in higher levels of crystallinity; the opposite effect is monitored at the long cooling time (Figure 8). This is explained by different opportunities for chain segments to undergo (a) formation of local well-ordered structures and (b) the relaxation of mechanical stresses. This finding is supported by the experimentally observed behavior [8,9].

It must be noted, however, that the simulations conducted here occur on a timescale that is extremely short compared to the real-life process. As a result, the strain rates are particularly high and the cooling times are very short. As previously detailed in an earlier publication [22], the observed effects are more pronounced. Specifically, it has already been demonstrated that crystallization effects are intensified due to strong orientations of chain segments [22]. Future investigations at lower strain rates may help to better relate the results obtained here to the actual process.

Nevertheless, in conclusion, this study underscores the complex nature of the crystallization of long polyethylene chains. By clearly revealing the interplay of different biaxial stretchings, orientation, entanglement, and crystallization behavior, we regard our methodology as a viable scientific approach. As we look ahead to the near future, it is our hope to extend the findings presented in this paper to polydisperse systems of different types of polymers. Finally, we would like to highly encourage other groups to explore the behavior of polymers in specific real-life processes through the application of MD methods.

## 4. Simulation Methodology

### 4.1. Force Field

For consistency reasons, we used the coarse-grained polyethylene force field [33] from previous publications [22,31,34], where three consecutive CH_n_ units are mapped into one superbead. A detailed description of the methods used for the development of the coarse-grained model is given in [33]. The force field is basically optimized to have good agreement with the experimental density and heat of vaporization.

As the equilibration of polymer melts is a highly demanding task, we point out that we paid special attention to the necessary procedures. The equilibration process is based on the findings of Auhl et al. [35] and Moriera et al. [36]. The procedure involves placing the chains into the cubic simulation box by a non-reversal random walk (NRRW) algorithm. The algorithm used is designed to ensure that the initial configuration of the chains has correct characteristic internal distances according to the freely rotating chain model [35]. The initial density is set to 0.75 g/cm³. Due to density fluctuations in the initial configuration, a zero-temperature Monte-Carlo (MC) algorithm is employed to minimize these fluctuations. A total of 150,000 MC move trials are conducted, treating the chains as rigid bodies, with each move that reduces fluctuations being accepted. Subsequently, during the warm-up phase, the non-bonded Lennard-Jones interactions are gradually introduced to prevent non-physical repulsive forces between overlapping particles. This stage has 6,500,000 time steps with a time increment of Δt=0.2 fs, using a Langevin thermostat (coupling constant 0.5 ps^−1^) to set the temperature to 500 K. Finally, the system is relaxed for 2,000,000 time steps (Δt=2 fs) by using a Berendsen thermostat ($\tau_{\mathrm{thermo}}=1\;\mathrm{ps}$, 500 K) and a Berendsen barostat ($\tau_{\mathrm{baro}}=1000\;\mathrm{ps}$, 1 bar). The procedure is described in full detail in [34]. With respect to the objectives of this study, systems of different sizes are equilibrated and further investigated (number of chains *M* × chain length *N*): 1000×500, 500×1000, 250×2000.

The results of the equilibration procedure are verified by monitoring the mean square internal distances and the static structure factor. These quantities have proven suitable for verifying the equilibration process [35]. For the purpose of validating the coarse-grained force field, it is reported that the systems have a density of the amorphous phase at 293 K ($\rho_{\mathrm{amorph},293K}$), melt density ($\rho_{500K}$), coefficient of thermal expansion (CTE), glass transition temperature ($T_{g}$), and crystallization onset temperature ($T_{c,\mathrm{on}}$) in good agreement with experimental results [22].

### 4.2. Simulation Procedure

**Figure 11:**
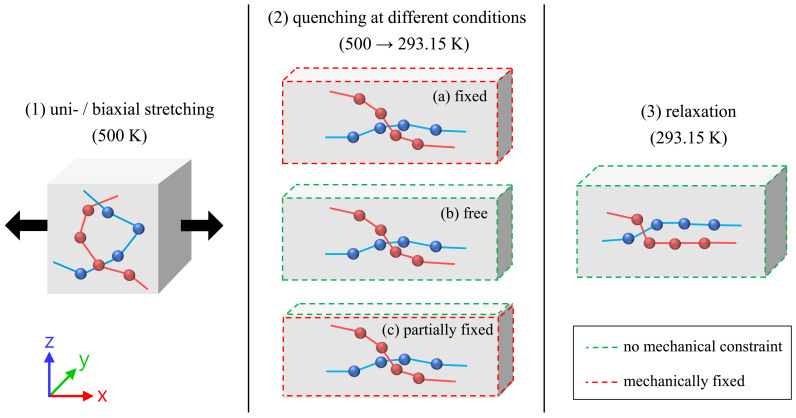
Of the three essential simulation steps: (1) uni-/biaxial stretching, (2) quenching, (3) relaxation. The mechanical boundary conditions used for the different mechanical states during quenching (2) are highlighted with different colors (free face (green), fixed face (red)). The differently colored spheres within the boxes represent exemplary superatoms, which belong to different polymer chains (red, blue). For reasons of clarity, the illustration of the stretching procedure (1) only demonstrates a uniaxial stretching in the x-direction.

The ESPResSo++ package (version 1.9.4.1) [37,38] was used to perform the molecular dynamics simulations. Starting with the equilibrated systems, the simulation procedure consists of three steps: (1) stretching of the samples of amorphous melt, (2) quenching of the samples to a specific temperature at three different conditions, (3) final relaxation at the target temperature. Figure 11 provides an overview of the individual steps as well as information on the boundary conditions.

In the first step, systems are continuously stretched in the melt state at 500 K. An initial strain rate of 1· 10^8^ s^−1^ is used. The stretching is performed by continuous deformation of the simulation box in corresponding directions. Systems are stretched up to different uni- and biaxial levels: in the case of uniaxial stretching, systems are stretched up to levels $\lambda_{\mathrm{uni}}=3$ and 6. In this study, these systems are referred to as “uni 3” and “uni 6”, respectively. Additionally, different biaxial levels of stretching ($\lambda_{x}$-$\lambda_{y}$) ranging from 2-2, 3-1.5, 3-2, 4-2 to 3-3 are used. In this case, systems are stretched simultaneously until the final level of stretching is attained. For the biaxially stretched systems, the initial strain rate of 1· 10^8^ s^−1^ refers to the main (larger) stretching direction. The strain rate of the minor stretching direction is adjusted so that the systems reach their respective final level of stretching simultaneously. The stretching is performed using the Berendsen barostat for the transversal directions ($\tau_{\mathrm{baro}}=10\;\mathrm{ps}$), Berendsen thermostat ($\tau_{\mathrm{thermo}}=1\;\mathrm{ps}$), and periodic boundary conditions (rectangular box).

The cooling of the samples is performed in three distinctively different ways. The first option is choosing (a) “fixed” boundary conditions, which do not allow for the previously stretched melt to contract after the final levels of stretching are attained. This allows for the internal relaxation of stress while the system (the simulation box) cannot change its size and shape. Simultaneously, the system is cooled down to the target temperature (293.15 K) within a time frame of 10 ns or, alternatively, 50 ns by using the Berendsen thermostat ($\tau_{\mathrm{thermo}}=1\;\mathrm{ps}$). This method is mainly used in this as well as our previous publications [22,31]. The second option is (b) “free” boundary condition: here, the box dimensions are allowed to change during cooling. This is performed by using an anisotropic Berendsen barostat ($\tau_{\mathrm{baro}}=1000\;\mathrm{ps}$) and setting the pressure to 1 bar in every direction of space. Temperature is controlled by the Berendsen thermostat ($\tau_{\mathrm{thermo}}=1\;\mathrm{ps}$). The third option uses (c) “partially fixed” boundary conditions by which we try to mimic real-life processing conditions especially with respect to the extrusion blow molding process closely [39]. In this case, the simulation box is fixed with respect to the plane of the previous stretching procedure. In the perpendicular (thickness) direction, the box is allowed to shrink during cooling. For this purpose, the Berendsen barostat is utilized following the definitions stated earlier. The Berendsen thermostat is applied as defined before as well. The integration time step for all simulation steps is set to 4 fs.

### 4.3. Evaluation of the Microscopic Structure

For the investigation of the orientation of bond vectors and chain end-to-end vectors, we refer to a widely established method [16,17,18,40]. The orientation factor $\delta_{\mathrm{bond}}$ is defined according to Equation (1) as the projection of the unit bond vectors ${\vec{e}}_{i}$, which connect consecutive beads that belong to the same chain, on the unit vector ${\vec{e}}_{\mathrm{testing}}$. The vector ${\vec{e}}_{\mathrm{testing}}$ is chosen such that it aligns with the specific direction of interest, e.g., the main or minor stretching direction. Brackets in Equation (1) indicate the average over all bond vectors.

(1) $$\delta_{\mathrm{bond}}=\frac{3}{2}\langle {\left({\vec{e}}_{i}\cdot {\vec{e}}_{\mathrm{testing}}\right)}^{2}\rangle -\frac{1}{2}$$

The orientation of chain end-to-end vectors $\delta_{\mathrm{end}-\mathrm{to}-\mathrm{end}}$ is evaluated analogously. For this purpose, the unit bond vector ${\vec{e}}_{i}$ in Equation (1) is substituted by the unit chain end-to-end vector ${\vec{e}}_{end-to-end}$. Additionally, the local ordering of chain segments is reviewed by the use of the nematic order parameter. The related nematic tensor can be expressed as

(2) $$Q_{\alpha \beta }=\frac{1}{N_{p}}\sum_{i=1}^{N_{p}}\left(\frac{3}{2}e_{i\alpha }e_{i\beta }-\frac{1}{2}\delta_{\alpha \beta }\right)$$

where $N_{p}$ is the number of evaluated bonds and α,β∈(x,y,z). The nematic order parameter *S* is the largest eigenvalue of this tensor. The local nematic order parameter $S_{\mathrm{local}}$ is determined for every bead *i* by evaluating all bonds found within a cutoff distance $r_{\mathrm{cut}}=2\sigma$ from the *i*th bead. For the entire system, we define ${\bar{S}}_{\mathrm{local}}$ as the mean value over all beads. The analysis is performed using the related implementation in the freud package (version 2.8.0) [41].

Entanglements of chains are evaluated by the primitive path analysis (PPA) according to Everaers et al. [42]. Details concerning the use of the PPA with respect to systems used in this study are given in [22]. On the basis of the PPA, the location of individual entanglement points is determined. The PPA straightens chains between fixed endpoints, while suppressing chain crossings due to the non-bonded interactions. Hence, entanglements show up as clear kinks [42]. We regard the location of these kinks as entanglement points. By the use of the geometric analysis according to Yashiro et al. [43], the location of the individual kinks and, hence, entanglement points are identified.

The level of crystallinity throughout this manuscript is calculated based upon the microscopic definition as described in [22]. The criterion evaluates the distance between non-bonded beads in conjunction with the orientation of corresponding bond vectors. If three or more consecutive beads ($n_{\mathrm{stem}}\geq 3$) within a chain are within appropriate threshold values, these beads are regarded as being in a crystalline state

## Data Availability

The data that support the findings of this study are available from the corresponding author upon reasonable request.
